# Prostate cancer screening: what do men know, think and do about their risk? exploring the opinions of men in an urban area in Lagos State, Nigeria: a mixed methods survey

**DOI:** 10.11604/pamj.2019.34.168.20921

**Published:** 2019-11-29

**Authors:** Uzoamaka Valerie Ugochukwu, Oluwakemi Ololade Odukoya, Augustine Ajogwu, Rufus Wale Ojewola

**Affiliations:** 1 Department of Community Health and Primary Care, College of Medicine of University of Lagos, Lagos State, Nigeria; 2 Department of Community Health and Primary Care Lagos University Teaching Hospital, Lagos State, Nigeria; 3 Department of Surgery, College of Medicine of University of Lagos/Lagos University Teaching Hospital, PMB 12003, Idi-Araba, Surulere, Lagos, Nigeria

**Keywords:** Prostate, cancer, screening, knowledge, attitude, prevalence, Nigeria, quantitative, qualitative, males

## Abstract

**Introduction:**

Prostate cancer (PCa) is the leading cause of cancer-related deaths in men aged 40 years and older. Incidence and mortality rates are higher in African men. PCa is amenable to early detection by screening which can prevent and reduce cancer deaths. Late-stage presentation and diagnosis often occur due to poor screening practices. This study assessed the knowledge, attitude, prevalence and barriers towards PCa screening among males in an urban area in Nigeria using a mixed method approach.

**Methods:**

This cross-sectional descriptive study used quantitative and qualitative methods among men aged 40 years and older. A pretested structured questionnaire was used to interview 344 respondents through multi-stage sampling. Additionally, two focus group sessions were held using a pre-tested guide.

**Results:**

Respondents were between 40-89 years with a mean age of 52.8 ± 9.9 years. Majority (54.9%) had poor knowledge of prostate cancer and its screening methods however, 65.7% expressed positive attitudes towards screening. Only 73 (21.2%) had ever been screened. The focus groups showed that respondents expressed a willingness to undergo PCa screening. The main barriers to screening were the fears of a positive result, ignorance and financial constraints. Participants preferred male physicians during digital rectal examinations.

**Conclusion:**

Respondents showed poor levels of knowledge. They expressed positive attitudes towards screening. However, this was not translated into practice. Public health interventions should educate men about benefits of early detection while addressing fears of positive findings and gender biases during rectal examinations. Efforts at providing low-cost alternatives for PCa screening are needed.

## Introduction

Prostate cancer (PCa) is the fourth leading cancer-related cause of death worldwide and the second most common cancer among men; an estimated 1.1 million men worldwide were diagnosed with PCa in 2012, accounting for 15% of the cancers diagnosed in men [1]. The burden of prostate cancer is expected to grow to 1.7 million new cases and 499,000 new deaths by year 2030. Various epidemiological data have supported the high incidence and mortality of this malignancy amongst the blacks [2]. Nigeria lacks a thorough cancer data base so information on PCa incidence are often based on individual reports [3]. In Nigeria, as with many black African countries, PCa is the most common cancer among males; in 2014, the World Health Organization (WHO) reported 30,400 cancer-related deaths in Nigeria and 31.7% of these deaths were as a result of prostate cancer [4]. In contrast to high-income countries, where mortality rate is low as a result of routine screening leading to early detection, majority of the cases in low and middle-income countries like Nigeria are diagnosed among symptomatic men at advanced stages with attendant higher mortality rates [5]. Assessing the knowledge, practice and uptake of screening methods among at-risk men in the community for PCa is a critical first step towards improving screening practices, early detection and treatment [6]. Prostate cancer screening is an attempt to diagnose PCa in asymptomatic men. This includes the measurement of serum prostate specific antigen (PSA) and digital rectal examinations (DRE). Despite the increased awareness of prostate cancer screening globally, the uptake has remained low especially in sub-Saharan African [6]. Qualitative methods of data collection are relevant in exploring knowledge and barriers to health-seeking practices in target populations [7]. Few studies have used qualitative methods to explore the knowledge, attitude and prostate cancer screening practices among males in countries where the burden of PCa is greatest [8]. We therefore used a mixed methods approach to assess the knowledge, attitudes and practices of PCa screening among a group of males in an urban environment in Lagos state.

## Methods

### Study setting, study design and study population

The cross-sectional descriptive study was carried out in Itire-Ikate local government, an urban area, in Lagos State, South-western region in Nigeria. The indigenes are predominantly of Yoruba ethnicity and are mostly petty traders, motorcycle riders, bus drivers and politicians. Ethical approval for the study was obtained from the Health Research and Ethics Committee of Lagos University Teaching Hospital. It was conducted among adult males aged 40 years and above. Only men who had resided in the community for at least six months and aged 40 years and above were included in the study.

### Quantitative data collection

#### Sample size, sampling method, data collection tools, techniques and data analysis

For the quantitative aspect of the study, the minimum sample size was calculated for the study using the standard formula for descriptive studies and based on the relevant findings of a previous study [5]. These findings revealed that only 28.4% of participants had ever tested for prostate cancer; this was used to calculate sample size for this study. Considering a confidence level of 95%, an alpha of 0.05 and a precision of 5% and an expected non-response rate of 10%, the final sample size for the study was 344. Respondents were selected using a multistage sampling method in five sequential stages i.e. selection of wards, streets, houses, households and respondents in that order. Quantitative data was collected by trained interviewers using a pre-tested questionnaire adapted from the following tools: The Thomas Jefferson University Prostate Cancer Screening Survey, The Knowledge of Prostate Cancer Screening Questionnaire, Knowledge and Practice of Prostate Health Questionnaire and Prostate Cancer Screening Education (PROCASE) Knowledge Index [9-11]. The occupational levels of respondents were classified using the International Standard Classification of Occupations [12]. Participation in the study was voluntary and a written informed consent was obtained prior to data collection.

Quantitative data was entered and analysed using SPSS version 21.0. Knowledge and attitude scores were computed and graded in the following manner. There were five (5) questions assessing the knowledge of prostate cancer and screening practices. Correct responses awarded one point and incorrect responses awarded zero point. These scores were summed and converted into a percentage scale. Levels of knowledge were categorized as poor (<50%) and good (≥ 50%). Attitude towards prostate cancer screening was assessed on a five-point Likert scale with the most positive response receiving five points and the most negative, one point. These scores were summed and converted into a percentage scale. Respondents with scores less than 50% were classified as having negative attitude, while those with scores of 50% or more had positive attitude. There were nine (9) questions assessing their attitude. Chi-square and t-tests were conducted to determine if there was any relationship between the respondents' socio-demographic variables, their knowledge, their attitude and prostate cancer screening. P values of < 0.05 were considered statistically significant.

### Qualitative data collection

Two focus group discussions (FGD) were conducted among a convenient sample of eight (8) adult males in each group; one group consisted of younger males i.e. 50 years and younger while the other consisted of older males i.e. 51 years and older. The focus groups were conducted at a neutral location within the community. Recruited participants were reminded of the date and venue of the sessions via phone calls on the previous day and the morning of the sessions. Each session began by welcoming the participants followed by an introduction of the research team with an explanation on their roles during the discussion. The topic and purpose of the discussion were explained and an informed consent obtained from each participant along with their demographic information such as age, occupation, educational level, and marital status. Each discussion was conducted by a trained researcher using a pretested FGD guide with participants seated in a semi-circular manner. Sessions were audiotaped while a research assistant took notes of the discussions, recurring statements and notable slangs. Each discussion lasted for about 55 minutes. The participants in both sessions were coded using the letters A-P. The qualitative data was analysed manually. The notes and audiotapes were transcribed verbatim. The focus group transcripts were read thoroughly to identify a coding structure that would provide a meaningful framework to capture respondents´ knowledge, attitude and barriers to prostate cancer screening.

## Results

**Quantitative results:** majority (54.9%) of the respondents had poor knowledge of prostate cancer screening and majority (65.7%) of them had a positive attitude towards prostate cancer screening (tables not included).

**Socio-demographic characteristics of the respondents:** majority (50.6%) of the participants were married. The majority (74.1%) of the participants were less than 60 years of age. Approximately 51% of the participants had only acquired a secondary school education and they accounted for the majority of the participants (Table 1).

**Table 1:** Socio-demographic characteristics of participants

Socio-demographic characteristics	Frequency (n=344)	Percentage
**Age group (in years)**		
40-49	153	44.5
50-59	102	29.6
60-69	58	16.8
70-79	26	7.6
>80	5	1.5
**Marital status**		
Married	242	70.3
Single	74	21.5
Divorced	8	2.3
Widowed	15	4.4
Separated	5	1.5
**Education level**		
Secondary	174	50.6
Not formal schooling	11	3.2
Primary	61	17.7
Tertiary	98	28.5
**Employment Status**		
Employed	312	90.7
Not employed	32	9.3
**Type of occupation (n=312)**		
Skilled	75	24.0
Semi-Skilled	218	69.9
Unskilled	19	6.1

**Knowledge of prostate cancer, screening methods and sources of knowledge:** television (25.6%) and radio (26.1%) were the most common sources of information on prostate cancer screening in participants. The number of respondents that believe it was possible to detect prostate cancer early was calculated at 48.8% (Table 2).

**Table 2:** Respondents knowledge of prostate cancer screening

Respondent is:	Frequency	Percentage
Aware that prostate cancer is preventable	265	77.0
Aware that prostate cancer is treatable if detected early	234	68.0
**Awareness of screening methods**		
Collection of blood sample (PSA test)	177	51.5
The doctor inserting a gloved, lubricated finger in my anus (DRE exam)	60	17.4
Don’t know	110	32.0
**Awareness of indications for screening**		
Positive family history	85	24.7
Older age (40 years and above)	160	46.5
Don’t know	110	32.0
**Primary sources of information about early detection (n=168)**		
Newspaper	43	25.6
TV	44	26.1
Doctor	29	17.3
Family/Friend	26	15.5
Internet	10	6.0
Other	16	9.5

DRE = Digital Rectal Examination. PSA = Prostate Specific Antigen

**Attitudes towards prostate cancer screening:** majority of the respondents have a positive attitude towards prostate cancer screening (65.7%). About two-fifth of the respondents (39.2%) strongly agree it is important to have a prostate cancer screening test. Slightly higher than one-third (37.5%) strongly agree that they are bothered by the possibility that DRE might be physically uncomfortable. Approximately 32% of the respondents strongly disagree that there are more important things to do than for prostate cancer screening (Table 3).

**Table 3:** Respondents’ attitude towards prostate cancer screening

Attitude Statement	Strongly DisagreeFrequency (Percentage)	DisagreeFrequency (Percentage)	NeutralFrequency (Percentage)	AgreeFrequency (Percentage)	Strongly AgreeFrequency (Percentage)
I am bothered by the possibility that DRE might be physically uncomfortable	50(14.5)	53(15.4)	31(9.0)	81(23.6)	129(37.5)
I am bothered by the possibility that a PSA test might be physically uncomfortable	133(38.7)	90(26.2)	36(10.4)	51(14.8)	34(9.9)
I am too busy to go for prostate cancer screening	109 (31.7)	112(32.6)	78(22.7)	28(8.1)	17(4.9)
It is important to me to have a prostate cancer screening test	23 (6.7)	21 (6.1)	37 (10.8)	128 (37.2)	135 (39.2)
I think men that have prostate cancer screening will have more problems than men who do not go for screening	117 (34.0)	72 (20.9)	77 (22.4)	50 (14.6)	28 (8.1)
I am afraid that if I have prostate cancer screening test, the test result will show that I have prostate cancer	97 (28.2)	92 (26.7)	69 (20.1)	69 (20.1)	17 (4.9)
I think going through digital rectal exam would be embarrassing to me	72 (20.9)	72 (20.9)	23 (6.7)	80 (23.3)	97 (28.2)
I think going through prostate specific antigen blood test for prostate cancer would be embarrassing to me	160 (46.5)	130 (37.8)	23 (6.7)	12 (3.5)	19 (5.5)

**Prostate cancer screening practices:** majority of the respondents have never been tested for prostate cancer (73.9%). However, respondents who had undergone screening using the PSA test were recorded at 80.8%. About 85% had tested negative for prostate cancer. More than one-third of the respondents had the test done because it was free (38.4%) (Table 4).

**Table 4:** History of prostate cancer testing, reasons for testing among those who have been screened and intentions to screen among the never tested

Respondent:	Frequency	Percentage
**Has ever been tested for prostate cancer**		
Yes	73	21.2
No	254	73.9
Not sure	17	4.9
**Time of most recent test (n=73)**		
More than 5 years ago	26	35.6
1-5 years ago	37	50.7
Less than a year ago	10	13.7
Type of screening test carried out (n=73)*		
Blood sample collection (PSA)	59	80.8
Digital rectal examination (DRE)	17	23.3
**Was informed of the outcome of the screening test (n = 73)**		
Yes	52	71.2
No	10	13.7
Can’t remember	11	15.7
**Reported outcome of the screening test (n=52)**		
Positive	5	9.6
Negative	47	90.4
Reasons for screening among those who had ever been screened (n=73) *		
Doctor’s request	18	24.7
I want to be healthy	24	32.9
It was free	28	38.4
Family member died of prostate cancer	12	16.4
Workplace requirement	1	1.4
Reasons for not screening among those who had never been screened (n= 271) *		
I don’t think I need it	52	19.2
The test is too expensive	50	18.4
I wasn’t aware I needed to be tested	74	27.3
Others	39	14.4
**Intention to screen within the next year (n=271)**		
Yes	140	51.7
No	74	27.3
Not sure	57	21.0

* Multiple responses allowed. PSA = Prostate Specific Antigen

**Bivariate analysis of the factors associated with knowledge, attitudes and screening practices:** most of the respondents with good knowledge were married (67.8%); had at least a tertiary level of education (43.4%) and had a semi-skilled occupation (66.4%).Men who were married (68.6%), semi-skilled (64.2%) and had at least a secondary school education (44.1%) had more positive attitudes towards prostate cancer screening. More men who were married (64.4%) had previously tested for prostate cancer (Table 5).

**Table 5:** Bivariate analysis of knowledge, attitude and prevalence of prostate cancer screening

Socio demographic variable	Poor knowledge (n=192) Frequency (Percentage)	Good knowledge (n=152) Frequency (Percentage)	P value	Poor attitudes (n=118) Frequency (Percentage)	Good attitudes (n=226) Frequency (Percentage)	P value	Ever screened (n= 73) Frequency (Percentage)	Never screened (n = 271) Frequency (Percentage)	P value
Age in mean (SD)	52.3(10.1)	53.3 (9.7)	0.349	52.6 (9.1)	52.9 (10.4)	0.835	59.5(10.7)	50.94(8.9)	0.000
**Marital status**			0.524			0.159			0.014
Married	139 (72.4)	103 (67.8)		87 (73.7)	155 (68.6)		47 (64.4)	195 (72.0)	
Single	37 (19.3)	37 (24.3)		26 (22.0)	48 (21.2)		14 (19.2)	60 (22.1)	
Divorced/separated/widowed	16 (8.3)	12 (7.9)		5 (4.2)	23 (10.2)		12 (16.4)	16 (5.9)	
**Ethnicity**			0.032			0.584			0.013
Yoruba	125 (65.1)	88 (57.9)		77 (65.3)	136 (60.2)		48 (65.8)	165 (60.9)	
Igbo	41 (21.4)	41 (27.0)		25 (21.2)	57 (25.2)		10 (13.7)	72 (26.6)	
Hausa	21 (10.9)	17 (11.2)		11 (9.3)	27 (11.9)		14 (19.2)	24 (8.9)	
Others	5 (2.6)	6 (3.9)		5 (4.2)	6 (2.7)		1 (1.4)	10 (3.7)	
**Highest level of Education**			0.000			0.137			0.075
Primary or less	50 (26)	22 (14.5)		25 (21.2)	47 (20.8)		21 (28.8)	51(18.8)	
Secondary	110 (57.3)	64 (42.1)		52 (44.1)	122 (54.0)		29 (39.7)	145 (53.5)	
Tertiary	32 (16.7)	66 (43.4)		41 (34.7)	57 (25.2)		23 (31.5)	75 (27.7)	
**Occupation**			0.059			0.932			0.041
Skilled	40(20.8)	35 (23.1)		25 (21.2)	50 (22.1)		13 (17.8)	62 (22.9)	
Unemployed	25 (13.0)	7 (4.6)		13 (11.0)	19 (8.4)		1 (1.4)	31 (11.4)	
Semi-skilled	117 (60.9)	101 (66.4)		73 (61.9)	145 (64.2)		53 (72.6)	165 (60.9)	
Unskilled	10 (5.2)	9 (56.9)		7 (5.9)	12 (5.3)		6 (8.2)	13 (4.8)	
**Religion**			0.003			0.859			0.782
Christianity	97 (50.5)	103 (67.8)		71(60.2)	129(57.1)		44 (60.3)	156 (57.6)	
Muslim	92 (47.9)	49 (32.2)		46 (39.0)	95 (42.0)		28 (38.4)	113 (41.7)	
Traditional	3 (1.6)	0 (0.0)		1 (0.8)	2 (0.9)		1 (1.4)	2 (0.7)	

### Qualitative results

Participants in both group discussions were aged between 41-58 years. Majority (87.5%) of the participants had at least a secondary level of schooling and most were married (81.3%), of Yoruba ethnicity (81.3%) and practiced Christianity (75%).

### Understanding of prostate cancer

The participants had some knowledge of cancer and the symptoms of prostatic enlargement:

*“Prostate cancer, it has to do with one´s urination. I had a friend whose dad died from prostate cancer, overtime he could not help himself so they had to put pipe and over time he died so it is that growth that makes your bladder expand overtime so it can´t contain the urine.”* (B, 56).

*“Prostate cancer, heard of it on Wazobia (an FM radio station); not sure exactly what it is, would like to know more”.* (I, 47).

### Early detection

Majority of the participants said that prostate cancer can be prevented and they felt that orthodox medical practitioners were best at diagnosing prostate cancer; some acknowledged screening as a means of early detection.

*“Once it is detected early and you know the symptoms, the doctor can tell you if you have it, if you do, he can tell you if it´s what can be controlled then it can be cured.”* (D, 50).

*“If it is detected, your doctor can counsel you on how to go about it”.* (H, 50)

### Attitudes towards prostate cancer screening

The participants stated that most people in their age bracket would feel apprehensive about being tested for prostate cancer for fear of receiving a positive result. When asked how they would feel about undertaking a prostate cancer screening test, two participants said:

*“I will feel nervous at first but will summon courage cos if anything is found then treatment will proceed; it is better to go than not at all”* (J, 45).

*“Happy, because for me, prostate cancer starts at an old age and at my age I would love to go if asked; even without anyone asking cos I already have mind to go for the test”.* (E, 55).

### Screening methods

#### Digital rectal examination (DRE)

The participants´ main concern about a DRE was about the gender of the health practitioner carrying out the procedure; however, the importance of the test could outweigh their concerns about the gender of the practitioner. They stated that they would feel ashamed if a female physician carried out the procedure on them.

*“Whatever the doctor instructs me to do, I will do. But if it´s a female doctor, I will feel ashamed but at the end I must do it so that the screening can be carried out. If it´s a male, I won´t be ashamed cos it´s a man like me but if it´s female, I will feel ashamed but I will do it because I have to”* (M, 44).

*“I will do the test but I must ask for man first. If there´s no man I will manage and do it”.* (F, 58)

### Prostate specific antigen blood test

Their major concern about the PSA was the associated pain of the needle prick and possible swaps in blood samples based on human errors. Otherwise they seemed to be comfortable with this screening method; no cultural or religious issues were raised about the PSA.

*“I won´t mind, except for the pain of the needle”.* (L, 48)

*“I will only follow to see; I don´t want them to exchange my blood”* (O, 42).

### Experiences with prostate cancer patients

Some of the respondents had experience with patients’ pain and symptoms as a result of prostate cancer:

*“My dad had prostate cancer, when he urinates, it doesn´t flow well. It is not easy for him, sometimes they connect pipe and recommend medicine. I know he felt pain especially when urinating because it does not flow freely as it did before. The situation was not easy for him until his death. It was not a smooth experience, I felt bad”.* (J, 45).

*“My friend who died in 2004. I stayed with him in the hospital, he carried pipe inside his body. He died from prostate cancer”.* (B, 56).

### History of screening

None of the participants had ever gone for prostate cancer screening. They attributed this to poor knowledge and attitude towards screening and a lack of awareness for the need of a screening test, due to the absence of symptoms. They also said that they did not know where to go for such a test.

*“No but I´d love to go for test because I know my ‘machine gun' is very active. I haven´t gone for the test because I have not seen any symptoms on me. If I see changes, I will go for the test. If my doctor says you have to go for the test and if I know where to go, I will go”.* (N, 45)

*“No, because I do not know where to go for the test; can we do it today? I´d like to go for it”.* (E, 55).

*“No, I have never gone for the test; I will attribute it to carelessness on my part. I just clocked 50 years, I have given testimony that I haven´t been hospitalized in my life so it´s just carelessness and I felt for 50 since I haven´t been hospitalized before that I´m sound but soon I will go”.* (H, 50).

### Perceived benefits of screening

All participants believed there were benefits to prostate cancer screening. They believed early detection was the major benefit to being tested for prostate cancer.

*“Yes, because at our age it is important to go for the test, to know what is in our bodies”.* (E, 55).

*“Yes, it´s always good to go to test so you can find out if there´s anything earlier”.* (K, 41).

### Barriers to screening

There were three prominent barriers: (fear, ignorance and financial constraints) to prostate cancer screening.

*“Ignorance is one reason and then some people that know are scared to go cos they feel that when you get the problem then you won´t have the money to cure the problem”.* (B, 56).

*“HIV/AIDS is now free cos now people volunteer themselves for the test so prostate cancer test should be free; the drugs are too expensive to buy so that makes people not to test, that´s why people don´t go. They would rather live with it until they die”.* (D, 50)

### Suggested ways of improving screening rates among men

Majority of the participants in the FGD suggested a focus on the importance of life as a way of convincing people to test for prostate cancer; some said they would only approach persons with whom they had a personal relationship while others would not approach anyone unless they sought their advice. Some believed in leading by example.

*“The easy way to convince people is through seminars; we will then be able to pass the message to everyone that may or may not know. Once you pass the message and let them know the importance, they will now know it´s a matter of life and death. It will be best to tell them how expensive or cheap it is so that way, they can make their own judgment”* (D, 50).

*“You can´t just tell anyone to go for the test except you come to me as a friend or someone you can ‘lick´ your secret to, that´s when the person will tell me. You might see someone and want to advice but then the person will be annoyed with you so I will wait for the person to talk to me about it then I can even follow you for the test”.* (J, 45)

*“I will go for the test first before trying to convince someone. If I haven´t done it, I can´t tell someone to do it so if I go first, I can willingly tell someone”.* (N, 45).

## Discussion

A key finding observed from the quantitative and qualitative aspects of this study was that majority of the respondents had never been screened for prostate cancer (73.9%) [13]. This low screening rate was also observed in a similar study in southwest Nigeria where only 10.2% of the respondents had ever been screened for prostate cancer. Similar findings were also observed among men in Ghana where 90% of the respondents had never been screened [14]. Considering the fact that prostate cancer is amenable to early detection, screening is critical for the early identification of cases and a reduction in prostate cancer deaths. The poor screening practices observed in this study may be as a result of respondents´ lack of awareness, low level of risk perception or financial barriers. Low levels of risk perception and poor awareness were also implicated as possible reasons for low screening rates among the male teachers in Nsukka, Eastern Nigeria and in Ghana [14, 15]. Poor levels of knowledge have also been cited as factors contributing to poor screening practices in similar studies among African American men in the USA [16, 17].

Among those that were reported to have been screened for prostate cancer, a greater number of them were screened during a free community health service in the locality (38.4%). Also, among respondents who had not screened, both the qualitative and quantitative findings suggested that many of the respondents felt screenings were expensive. Similar findings have also been observed in developed countries like the USA, in situations where screening is not covered by standard health insurance packages [16]. This may highlight the need to consider the inclusion of free or subsidized community screening services into existing primary health care structures or to promote the inclusion of routine prostate cancer screening in basic health insurance packages.

Only (24.7%) were screened based on advice by a health care practitioner suggesting that missed opportunities for health education and screening may have occurred as only one in four men were screened because they received advice from their physician. Concern for possible missed opportunities and adequate pre-screening patient education have also been raised in the USA. The 2017 American Cancer Society prostate cancer screening guidelines emphasize that screening for prostate cancer should only occur after a detailed discussion about the known risks and benefits involving the patients; especially those at elevated risk of prostate cancer in a shared decision making [18].

In this study, although screening rates were poor, it was observed that respondents were nevertheless willing to be screened, as slightly more than half of the men who had never been screened (51.7%) indicated an interest in screening within the next year. Positive attitude and willingness to undergo screening have also been reported among men in Ekiti state and in Nsukka which are located in the South-Western and South-Eastern parts of Nigeria respectively [13, 15].

The fear of a possibly positive test results was noted as a barrier to future screening in the qualitative aspect of this study. This fear has also been expressed by respondents in previous studies [15,17]. In the study among male employees of the University of Nigeria, more than half of the respondents did not want to be screened because of the fear and anxiety associated with a possible positive result [15]. Lack of adequate knowledge may create fear and anxiety which increases the likelihood that an individual will not access information on prevention [16]. Interestingly, the fear of positive results has been a common finding in cancer screening generally. For instance, in a critical review study on fear, anxiety, worry and breast cancer screening behaviour, it was discovered that women´s primary fears surrounding breast cancer and its screening was the fear of a positive diagnosis, in addition to the fear of pain/discomfort associated with testing [19].

Some men may find the idea of having a digital rectal examination (DRE) uncomfortable. However, clear gender preferences for DRE, in favour of male health practitioners were noted in the study. Several community-based studies have cited DRE as one of the barriers to screening [14, 16]. In contrast; the study in University of Nigeria among male employees who may be more educated reported that DRE was not a barrier to screening [15].

A key strength of this study is the fact that it is one of the few studies that has assessed the knowledge, attitudes and screening practices of prostate cancer using both qualitative and quantitative methods. The findings may however need to be interpreted with some caution as it also has some limitations. Firstly, data was collected by self-report and is prone to misreporting and recall bias. Secondly, causal inferences cannot be made as the study is cross sectional in nature. Further research is needed to understand how best to address the main barriers to screening and to promote adequate pre-screening counselling. In addition, research on the psychological and emotional influences of the acceptability of preventive services in the population is also warranted.

## Conclusion

This study showed that although the knowledge of prostate cancer screening among the men were poor, they expressed positive attitudes towards screening. Their willingness to go through with the screening was however subject to certain conditions like subsidized costs and a preference for examinations by male physicians.

### What is known about this topic

- Prostate cancer is the leading cause of cancer related deaths among African men and the incidence increases with advancing age;
- Screening leads to early detection and reduced mortality among men.

### What this study adds

- Prostate cancer screening is low among this group of older men. However, opportunities for screening may be welcome as many of them expressed positive attitudes towards screening, particularly if it is free or subsidized;
- Pre-screening counselling to address the fears of a possibly positive result might be helpful.

## Competing interests

The authors declare no competing interests.
